# Safety of high-dose-rate stereotactic body radiotherapy

**DOI:** 10.1186/s13014-014-0317-0

**Published:** 2015-01-23

**Authors:** Sonja Stieb, Stephanie Lang, Claudia Linsenmeier, Shaun Graydon, Oliver Riesterer

**Affiliations:** Department of Radiation Oncology, University Hospital Zurich, Rämistrasse 100, 8091 Zurich, Switzerland

**Keywords:** Flattening filter free, Stereotactic body radiotherapy (SBRT), Stereotactic ablative radiotherapy (SABR), Dose rate, Toxicity

## Abstract

**Background and purpose:**

Flattening filter free (FFF) beams with high dose rate are increasingly used for stereotactic body radiotherapy (SBRT), because they substantially shorten beam-on time. The physical properties of these beams together with potentially unknown radiobiological effects might affect patient safety. Therefore here we analyzed the clinical outcome of our patients.

**Material and methods:**

Between 3/2010 and 2/2014 84 patients with 100 lesions (lung 75, liver 10, adrenal 6, lymph nodes 5, others 4) were treated with SBRT using 6 MV FFF or 10 MV FFF beams at our institution. Clinical efficacy endpoints and toxicity were assessed by Kaplan-Meier analysis and CTCAE criteria version 4.0.

**Results:**

Median follow-up was 11 months (range: 3–41). No severe acute toxicity was observed. There has been one case of severe late toxicity (1%), a grade 3 bile duct stricture that was possibly related to SBRT. For all patients, the 1-year local control rate, progression free survival and overall survival were 94%, 38% and 80% respectively, and for patients with lung lesions 94%, 48% and 83%, respectively.

**Conclusions:**

No unexpected toxicity occurred. Toxicity and treatment efficacy are perfectly in range with studies investigating SBRT with flattened beams. The use of FFF beams at maximum dose rate for SBRT is time efficient and appears to be safe.

## Background

Novel linear accelerator technology allows compensating non uniform photon fluence by use of intensity modulation instead of directing the beam through a flattening filter. A major advantage of using flattening filter free (FFF) beams is that the dose rate can be multiplied in comparison to flattened beams thereby shortening beam-on time [1,2]. This is especially attractive when a high dose per fraction is used, e.g. in the case of stereotactic body radiotherapy (SBRT). Although FFF beams at maximum dose rate have been rapidly introduced into clinical use, little clinical data about their safety and efficacy are available.

Shortening of treatment time improves patient comfort especially in elderly and frail patients and was shown to improve patient stability [2-4]. On the other hand, it may introduce novel hazards, e.g. in case of patient or organ movement there is less time to intervene. There are also dosimetric uncertainties due to the interplay effect; e.g. Ong et al. [5] investigated the dosimetric impact of intrafractional motion during spine radiosurgery and found an increased sensitivity of the target coverage and dose to the spinal cord if FFF beams are used compared to flattened beams.

Because of putative unknown radiobiological hazards of using high instantaneous dose rate, several research groups recently performed preclinical investigations. Using exclusively *in vitro* assays, four studies could not detect any effect of high dose rate on clonogenic cell survival [6-9], whereas Lohse et al. [10] found that FFF beams given with high dose per pulse impaired clonogenic survival and this effect became significant with high dose per fraction. Only one preclinical study so far investigated the effect of extremely high dose rate *in vivo*. Using a murine lung fibrogenesis model as well as human xenografts and murine syngenic and orthotopic lung tumors a differential response between normal and tumor tissue in mice was shown when ultrahigh-dose-rate flash irradiation was compared to conventional dose rate irradiation [11]. Also classical radiobiological considerations seem to support a dose rate effect of very high dose rates. Based on the linear quadratic model, in a recent comprehensive review it was suggested that the influence of high dose rate on tumor response and toxicity is determined by beam-on time with late reacting tissue being more sensitive to dose rate effect than tumors and early reacting tissues [12].

Our clinic was the first to use FFF linear accelerator technology at maximum dose rate for SBRT in patients. With the lack of a clear biological rationale not to use FFF beams we introduced this technology assuming that the benefit in terms of increased treatment efficiency would outweigh the minimal risk of additional toxicity. Here we report the results of our patients with focus on patient safety, which is the biggest patient cohort reported so far.

## Methods

From March 2010 to February 2014 84 patients were treated with SBRT using FFF beams at our institution. Patient and treatment parameters are listed in Table 1. Clinical data were collected retrospectively from the patient charts and radiotherapy parameters were extracted from the Eclipse treatment planning system.

**Table 1:** **Patient and treatment parameters**

	**N** ^**1**^
*Gender*	
Male	48 (57%)
Female	36 (43%)
*Age*	
Mean/Median [years]	66/67
Range [years]	19 - 88
*RT localization*	
Lung	75 (75%)
Liver	10 (10%)
Adrenal gland	6 (6%)
Lymph node	5 (5%)
Other site	4 (4%)
*Lesions treated*	
Primary tumor	25 (25%)
Metastasis	45 (45%)
Tumor recurrence	16 (16%)
Primary tumor/Metastasis	14 (14%)
*Prescription dose*	
48 Gy/4 fx	19 (19%)
40 Gy/4 fx	10 (10%)
35 Gy/5 fx	6 (6%)
60 Gy/10 fx	5 (5%)
50 Gy/10 fx	5 (5%)
Other	55 (55%)
*BED*	
<100 Gy	66 (66%)
≥100 Gy	34 (34%)
*GTV size*	
Mean [cm^3^]/Median [cm^3^]	15.4/6.7
Range [cm^3^]	0.60 - 126.7
<14 cm^3^	65 (65%)
≥14 cm^3^	35 (35%)
*PTV size*	
Mean [cm^3^]/Median [cm^3^]	58.2/35.2
Range [cm^3^]	5.9 - 368.7
*Energy*	
6 MV	84 (84%)
10 MV	16 (16%)
*Dose rate*	
Mean [MU/min.]/Median [MU/min.]	1319/1400
Range [MU/min]	451 - 2400

N^1^ = number of patients or lesions if not indicated otherwise. (RT: Radiation Therapy, fx: fractions, BED: Biologically Effective Dose, GTV: Gross Tumor Volume, PTV: Planning Target Volume, MU: Monitor Unit).

### Patients and lesions treated

Altogether 84 consecutive patients with 100 lesions were included in this study. Inclusion criteria were defined as the use of FFF beams at maximum dose rate for SBRT. Patients with pancreatic lesions were excluded due to the pre- or postoperative setting of radiotherapy. Of the 84 patients, 48 were male and 36 female. Patient age ranged from 19 to 88 years (median: 67 years).

The irradiated lesions were localized in the lung 75 (75%), liver 10 (10%), adrenal glands 6 (6%), lymph node 5 (5%) or other sites 4 (4%) (Table 1). Of all tumors 25% were primary tumors (19 non-small cell lung cancers and 2 small cell lung cancers with histology, 2 solitary lung nodules suspicious for primary lung cancer but without histology, 2 liver cancers), 45% metastases, 16% tumor recurrences and for 14% no differentiation was possible. Mean gross tumor volume (GTV) was 15.4 cm^3^ (range: 0.6–126.7 cm^3^). BED was ≥100 Gy in 33% of the patients. For patients with lung lesions the mean forced expiratory volume (FEV1) was 1.87 l (range: 0.41–4.30 l) and the mean diffusion capacity (DLCO) was 66% (24–115%).

### Treatment

A TrueBeam linear accelerator (Varian Medical Systems, Palo Alto, CA) was used to apply 6 MV FFF or 10 MV FFF beams with a maximum dose rate of 2400 MU/min. Overall mean dose rate considering all treatments was 1319 MU/min (range: 451–2400 MU/min). Patients were treated using Volumetric Modulated Arc Therapy (VMAT) with 1–4 arcs. The tumors were delineated as internal target volumes (ITV) on 4-dimensional computed tomographies (4DCTs). For peripheral and central lung lesions the planning target volume (PTV) was defined as the ITV + 6 mm and 3 mm, respectively, and for liver, adrenal gland and lymph node lesions ITV to PTV margins of 5 – 10 mm were used. Prescribed doses were 21–66 Gy in 3–15 fractions. In case of curative intention a BED of at least 100 Gy was envisaged (usually 48 Gy in 3 to 4 fractions, 10 × 6 Gy for tumors adjacent to the thoracic wall, 8 × 7.5 Gy for centrally located lung tumors). For palliative indications individualized and risk-adapted regimens were used depending on tumor localization, remaining organ function and anticipated life expectancy. Normal tissue constraints for lung tumors were adopted from current RTOG protocols (RTOG 0618 and 0915, http://www.rtog.org). Treatment planning was performed on the average projection of the 4DCT in the Eclipse treatment planning system (Varian Medical system, Palo Alto). The dose was prescribed inhomogeneously to the PTV in a way that 95% of the PTV should receive 100% of the prescribed dose with maximum doses between 125% to 152% [13].

### Follow up

The patients were usually seen at three months after SBRT and then in 6-monthly intervals. For follow-up imaging either CT of the chest or FDG PET/CT were used.

### Data analysis

Local control rate (LCR), progression free survival (PFS) and overall survival (OS) were assessed at 1 year after start of treatment. Local tumor recurrence or distant relapse were evaluated following the RECIST criteria version 1.1 [14]. In case RECIST criteria were not useful, an FDG PET/CT was performed to differentiate between tumor recurrence and lung fibrosis. Acute and late toxicities were assessed according to the Common Terminology Criteria for Adverse Events Version 4.0 (CTCAE v4.0). Acute toxicity was defined as adverse event within a 90 days period from start of treatment and late toxicity as adverse event thereafter. Grade 3–4 toxicity was assessed for all patients and grade 1–2 toxicity only for radiological changes in the lung (pneumonitis, pleural effusion, atelectasis) due to the retrospective character of the analysis. The biologically effective dose (BED) was calculated by the following formula: $\mathrm{BED}=nd\left(1+\frac{d}{\alpha /\beta}\right)$, whereby *α*/*β* was assumed to be 10 (n: number of fractions, d: dose per fraction). BED was calculated and analyzed for prescribed dose.

### Statistics

Descriptive statistics were used to calculate number, percentage, mean, median and standard deviation. Survival curves were calculated by Kaplan Meier analysis. Log-rank test and Cox regression were used to analyze correlation of outcome with BED, tumor size and mean dose rate. For log-rank test a BED of 100 Gy and a tumor volume of 14 cm^3^ (diameter ~ 3 cm) were chosen as cut-off values because BED above 100 Gy and tumor size ≤ 3 cm were associated with improved tumor control rates after SBRT with flattened beams [15-20]. A p-value of ≤ 0.05 was considered to be statistically significant. All statistical analyses described above were performed using the software program SPSS Version 22 (IBM SPSS Statistic Software).

## Results

### Toxicity

There was no treatment related mortality or ≥ grade 3 acute toxicity observed. Only 2 patients (3%) presented with grade 2 acute pneumonitis (Table 2).

**Table 2:** **Acute and late lung toxicity in 75 patients with lung lesions**

**Adverse event**	**Grade**		
	**I**	**II**	**≥III**
*Acute toxicity*			
Pneumonitis	11%	8%	-
Pleural Effusion	3%	-	-
Atelectasis	8%	-	-
*Late toxicity*			
Pneumonitis	44%	6%	-
Pleural Effusion	14%	2%	-
Atelectasis	20%	2%	-

If patients were treated at multiple sites, each site was analyzed separately.

Late toxicity could be assessed in 74 of 84 patients, who had a minimum follow-up of 90 days from start of treatment. One patient with centrally located liver metastasis developed a grade 3 bile duct stenosis after SBRT with 4 × 12 Gy. However this patient also had hemihepatectomy after SBRT with the resection line being close to the bile duct and the relationship to both treatments remains unclear. The patient received a percutaneous biliary drainage and was still alive at last follow-up, 22 months after treatment. Of 75 lung lesions 63 were peripherally and 12 centrally located. No ≥ grade 3 late lung toxicity occurred and there have been only 4 patients (5%) with grade 2 late lung toxicity: two cases with pneumonitis, one case with pneumonitits and atelectasis and one case with pleural effusion.

### Treatment efficacy

**Figure 1:**
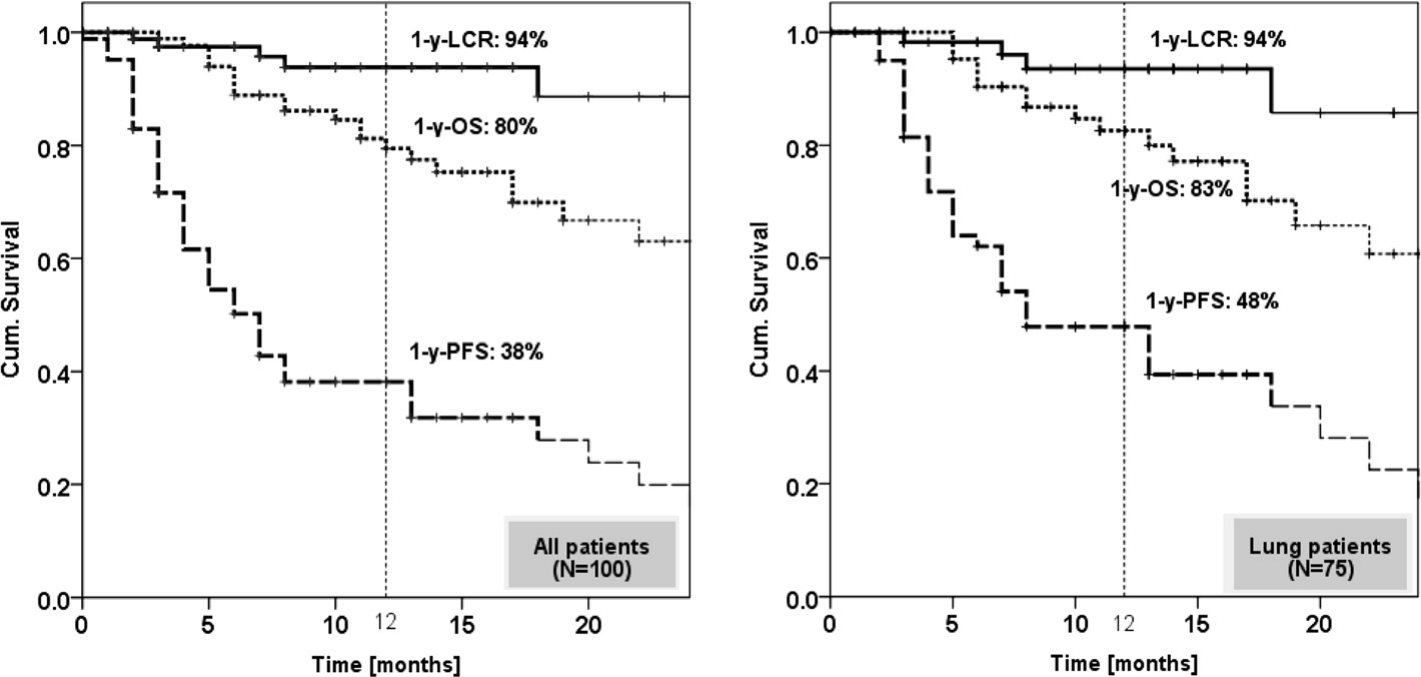
**Kaplan Meier curves for local control rate (LCR), overall survival (OS) and progression free survival (PFS) for all lesions (left side) and separately for lung lesions (right side).**

The median follow-up of all patients was 11 ± 10 months. For all patients the 1-year LCR, PFS and OS were 94%, 38% and 80% and for patients with lung lesions 94%, 48% and 83%, respectively (Figure 1). If lung lesions are stratified into primary lung cancers (n = 48) and lung metastases of other histologies (n = 27) the 1-year LCR, PFS and OS were 93%, 60% and 85% for primary lung cancers and 95%, 29% and 77% for lung metastases. All patients with liver metastases (n = 9) or thoracic lymph nodes (n = 4) remained locally stable after one year and all but one patient with adrenal lesion (n = 5). No significant correlation could be found between GTV size and LCR, if all lesions were analyzed, whereas for lung lesions there was a significant negative correlation when using the log-rank test with a cut-off value of 14 cm^3^ (p = 0.02), but not if Cox regression analysis was performed (Table 3). No significant correlation was found for LCR and the PTV surrounding BED (p = 0.52), but all patients irradiated with BED ≥ 100 Gy remained locally stable after 1 year of treatment, or between LCR and mean dose rate (p = 0.44).

**Table 3:** **Local Control Rate (LCR) 12 months after start of radiotherapy for all lesions and lung lesions**

		**All (N = 100)**		**Lung (N = 75)**	
**LCR – 12 months**	All	93.8% (N = 83)		93.5% (N = 61)	
	BED ≤ 100 Gy	90.7% (N = 55)	p = 0.43	89.7% (N = 39)	p = 0.51
	BED > 100 Gy	100% (N = 28)		100% (N = 22)	
	GTV ≤ 14 cm^3^	98.1% (N = 58)	p = 0.06	97,9% (N = 51)	p = 0.02
	GTV > 14 cm^3^	84.9% (N = 25)		74.1% (N = 10)	
**Cox regression**	BED	p = 0.36 (0.994)		p = 0.21 (0.986)	
	GTV	p = 0.72 (1.013)		p = 0.16 (0.986)	

p-values indicate significance determined by log-rank test. Cox Regression is shown with p-values and hazard ratio in brackets. (BED: Biologically Effective Dose, GTV: Gross Tumor Volume).

## Discussion

In the present study we analyzed the clinical results of the patients treated with SBRT and FFF beams at maximum dose rate at our institution. We included all patients treated so far, although it is a hetergenous cohort, because we wanted to analyze overall patient safety and detect any unexpected toxicity rather than investigating specific tumor sites. We show in 84 patients with 100 lesions in the lung, liver, adrenal glands, lymph nodes and other sites that the use of FFF beams for these indications is associated with low rates of acute and early late toxicity and results in excellent early local tumor control. The results shown appear similar to results of studies using SBRT with flattened beams and lower dose rates.

Most of the data published about FFF beams explored their physical properties [1,2,13,21-25]. Altogether these studies show that the use of FFF beams significantly reduces treatment time for high single fractions and dose distributions are comparable to flattened beams. Apart from the technical benefits there have been some concerns about patient safety when FFF beams with high instantaneous dose rate are used due to physical uncertainties of beam application and biological considerations.

A physical concern is that very short beam-on times introduce dosimetric uncertainties in case of tumor shifts. In patients with spinal metastases Ong et al. [5] investigated shifts of 1–5 mm during 5–30 seconds in the case of single fraction SBRT with flattened or FFF beams. Dosimetric deviations in FFF plans were approximately 2-fold greater than with flattened beams, which resulted in significant overdosage of the spinal cord and underdosage of the GTV with increasing shift size and dependent on shift duration. In their study GTV to PTV margins of 2–3 mm were used. In our clinic until recently we did not use SBRT for spinal metastases and therefore the lesions analyzed had usually less proximity to critical organs. In addition we fractionated SBRT und applied much larger margins (3–10 mm). Therefore potential dosimetric uncertainties are much less important for the treatments reported by us.

Recently several preclinical studies were published investigating the biology of high instantaneous dose rate in clonogenic cell survival assays [6-10]. Sorensen et al. used Chinese hamster and FADU cells irradiated with 6 MV flattened beams with single doses up to 10 Gy, dose rates between 5 and 30 Gy/min and instantaneous dose rates per pulse between 56 and 338 Gy/s [8]. They did not find any effect of high instantaneous dose rates on cell survival. In contrast, Lohse et al. [10] irradiated different glioblastoma cell lines using 10 FFF beams with overall dose rates of 0.2 Gy/min or 24 Gy/min and instantaneous dose rates per pulse up to 350 Gy/s and found that high dose per pulse but not delivery time reduced clonogenic survival. The anti-tumor cell effect of high dose rate increased with fraction size and became significant at 10 Gy. Three additional preclinical studies were published investigating FFF beams in different cell-lines, all showing no difference in clonogenic cell survival if FFF beams with dose rates up to 24 Gy/min are used [6,7,9]. A preclinical study using clinically more relevant *in vivo* models was recently published by Favaudon et al. [11]. The authors used a lung fibrogenesis model and human tumor xenografts as well as syngenic and orthotopic murine lung tumors and treated them with high single fractions of ultrahigh-dose-rate flash irradiation (dose rate ≥ 40 Gy/s) versus conventional dose rate irradiation (≤0.03 Gy/s). Interestingly both techniques exerted similar anti-tumor effects but the ultrahigh-dose-rate flash irradiation induced much less TGF-β dependent lung fibrosis indicating a differential response between normal and tumor tissue. A somehow contrary differential effect was recently suggested and modeled by Ling and coauthors considering various factors such as different mechanisms of DNA repair and implications of the α/β model [12]. Their conclusion was that the dose rate effect is not determined by the instantaneous dose rate but instead by beam-on time, which mostly affects late reacting tissues. By computing with the LQ Model they calculated for example that, if a single fraction of 10 Gy is given in 2 minutes instead of 10 minutes, this acceleration increases BED to the tumor (α/β = 10) by ~1.5% and BED to late responding tissue (α/β = 3) by ~3.9%.

We previously demonstrated that the gain in beam-on time by using 6 or 10 FFF beams in comparison to a 6 MV flattened beam increases with dose per fraction and amounts to several minutes when fractions beyond 10 Gy are used [2]. Therefore based on the paper by Ling et al. there might be clinical situations, e.g. in case of spinal SBRT when high doses are given in close proximity to critical serial organs, when the use of FFF beams with maximum dose rate might lead to a small increase of complications.

Only few clinical studies so far investigated the clinical outcome of SBRT with FFF beams in patients [26-31] (Table 4). Almost all studies except the studies by Prendergast and Wang were conducted from the research group of the Humanitas Cancer Center in Milano [26-28,30,31]. Generally, patient numbers in these studies were small and the follow-up was short. In the biggest retrospective patient series, 67 patients with 70 lesions in different organs were treated with SBRT using FFF beams [30]. The treatment schedules ranged from 32–48 Gy in 4 fractions for lung and 75 Gy in 3 fractions for liver tumors. With a minimum follow-up of 3 months two acute grade 3 lung toxicities (3%) were observed and 89% of patients showed a tumor response at 60–90 days after SBRT. In the only prospective study published with FFF beams so far, 40 prostate cancer patients were treated with 35 Gy in 5 fractions [26]. After a median follow-up of 11 months no grade 3 toxicity was observed.

**Table 4:** **Clinical studies investigating toxicity and outcome of SBRT with FFF beams**

	**Current study**	**Wang 2014 [** 31 **]**	**Prendergast 2013 [** 29 **]**	**Alongi 2013 [** 26 **]**	**Navarria 2013 [** 28 **]**	**Alongi 2012 [** 27 **]**	**Scorsetti 2011 [** 30 **]**
**Patients (Lesions)**	84 (100)	20 (22)	64	40	46	25 (28)	67 (70)
**Tumor**	Various (mostly lung)	HCC	Lung	Prostate	NSCLC Stage I	Abdominal/pelvic LN	Various (mostly lung)
**RT schedule**	21 – 66 Gy (3–15 fx)	40 - 75 Gy (3–10 fx)	30 – 60 Gy (3–5 fx)	35 Gy (5 fx)	48 Gy (4 fx)	45 Gy (6 fx)	32 – 75 Gy (3–6 fx)
**Median follow-up (range)**	11 months (3–46)	7 months (3–13)	12 months (3–25)	11 months (5–16)	16 months (2–24)	6 months (2–19)	NA^7^
**Acute toxicity** ^**1**^ **≥ G3**	0%	5%^3,6^	2%^4^	0%	4%^4,6^	0%	3%^4^
**Late toxicity** ^**1**^ **≥ G3**	1%^2^		12%^4,5^	0%		0%	-
**Local control**	1-y-LC: 94%	Actuarial-LC: 95%	NA^8^	NA^8^	1-y-LC: 100%	Actuarial-LC: 100%	Actuarial-LC: 89%

^1^Toxicity was assessed either by number or by lesion; ^2^Bile duct stricture; ^3^Radiation induced liver disease (RILD); ^4^Pulmonary toxicity; ^5^Brachial plexopathy; ^6^Data show overall toxicity; ^7^Minimum follow up 3 months; ^8^Local control not reported. RT: radiotherapy, fx: fractions, LC: local control, LN: lymph node, y: year, NA: not applicable.

In the studies of the Milano group the dose was prescribed homogeneously to the 95% isodose [26-28,30]. In contrast, we use a SBRT protocol, where the dose is prescribed inhomogeneously to the PTV with dose maxima in the tumor of up to 152%. Therefore, in case of similar dose prescription, the patients reported by us received a substantially higher dose to the GTV and immediately surrounding PTV without evidence of increased toxicity. The only study that reported substantial high-grade toxicity when using SBRT with FFF beams was published by Prendergast et al. [29]. This study included 64 patients with lung malignancies receiving 30–60 Gy in 3–5 fractions. Median follow up was 11.5 months and 6 cases of severe ≥ grade 3 late toxicity (12%) occurred; of these, five were pulmonary and one nerve-related. One patient died because of sepsis after pneumonia in the irradiated lobe. Tumor control and survival data were not presented.

We report a 1-year local control rate of 94%. All tumors treated with a PTV surrounding BED > 100 Gy were locally stable after one year and small lung tumors ≤ 14 cm^3^ (~3 cm diameter) had a significant lower recurrence rate. Dependency of tumor control on BED and tumor size is well known from studies predominantly performed with flattened beams [19,32,33]. In addition, the toxicity reported by us is similar or even lower in comparison to the toxicity reported in the literature for SBRT with flattened beams [17,34-36]. In order to detect small changes in toxicity, when FFF beams are used, large randomized studies would be needed, which are unlikely to be performed, because FFF beams are rapidly adopted into the clinic and are FDA-approved. In addition to LCR and toxicity we also analyzed PFS and OS. Of clinical importance, the PFS was much lower than the LCR suggesting that patients tend to recur distant from the irradiated lesion. In addition, the low numbers of 1-year PFS and OS in primary and secondary cancers indicate a selection bias of inoperable, comorbid or extensively pretreated patients.

A limitation of our study is the retrospective design, which doesn’t allow evaluating all side effects of radiotherapy in the same manner. On the basis of medical records we often could not distinguish between toxicity grade 1 and 2, therefore we focused on side effects of grade 3 and higher: events that made hospitalization or prolongation of hospitalization necessary. Because of only one case of ≥ grade 3 toxicity we could not perform statistical analysis of toxicity related risk factors (e.g. radiation dose, tumor size, centrally located vs. peripheral lung tumors, etc.). Although our clinic was the first center to implement FFF beams for clinical use, the median follow-up of 11 months is relatively short with, on the other hand, the follow-up period ranging up to 41 months.

## Conclusion

In our cohort of patients with lung, liver, adrenal gland and lymph node lesions, who were treated with FFF beams, the acute and early late toxicity, local control and survival data are perfectly in range with the literature investigating SBRT with flattened beams. Therefore the use of FFF beams for SBRT appears to be safe for these indications, if carefully applied.
